# Primary Dissociated Midbrain Dopamine Cell Cultures from Rodent Neonates

**DOI:** 10.3791/820

**Published:** 2008-11-05

**Authors:** Lauren E. Frank, Angela D. Caldera-Siu, Emmanuel N. Pothos

**Affiliations:** Department of Pharmacology and Experimental Therapeutics, Tufts University

## Abstract

The ability to create primary cell cultures of dopamine neurons allows for the study of the presynaptic characteristics of dopamine neurons in isolation from systemic input from elsewhere in the brain.  In our lab, we use these neurons to assess dopamine release kinetics using carbon fiber amperometry, as well as expression levels of dopamine related genes and proteins using quantitative PCR and immunocytochemistry.  In this video, we show you how we generate these cultures from rodent neonates.

The process involves several steps, including the plating of cortical glial astrocytes, the conditioning of neuronal cell culture media by the glial substrate, the dissection of the midbrain in neonates, the digestion, extraction and plating of dopamine neurons and the addition of neurotrophic factors to ensure cell survival.

The applications suitable for such a preparation include electrophysiology, immunocytochemistry, quantitative PCR, video microscopy (i.e., of real-time vesicular fusion with the plasma membrane), cell viability assays and other toxicological screens.

**Figure:**
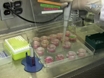


## Protocol

### Preparation Preceding the Culture

*Note*: ** Glial cells must be plated well in advance so that they have time to proliferate and cover the bottom of the  dishes. For mice with atypical or experimental genetic background, make sure to match glial and neuronal cultures in terms of genotype.

*Note*: ** 1-7 days before dissection, prepare fresh neuronal medium and replace the glial medium in the dishes with 2ml.

*Note*: ** On the day before dissection, the following items need to be left under UV light overnight:

- 4 dissection plates
- 2 dissociation vials with a micro-magnetic stir bar
- 2 vial caps (with 2 small holes poked in the top)
- Spread out slide rings (and coat in 70% EtOH) - leave the box open also
- AT LEAST 2 full boxes of yellow (10-200μl) pipette tips
- 1 box of blue (100-1000μl) pipette tips

### Day of Culture

*Note*: * BE SURE NOT TO TOUCH ANYTHING THAT WAS LEFT OUT FOR UV OVERNIGHT!Maintaining a sterile environment under the hood is very important.

#### 1) Set Up:

*Note*: ** Set aside all frozen ingredients to make papain solution.

1. Clean forceps with 70% EtOH. Cover each tip with a sterile yellow pipet tip. Use to place sterile slide rings in their box.
2. Hot plate:
  1. Place a mini-magnetic/hot plate under the hood with a 1000ml beaker filled to 500-600ml dH_2_O and a large magnetic stir bar.
  2. Place a Styrofoam disk in the beaker just above water level.
  3. Slide the thermometer in a small hole in the Styrofoam disk. Heat dial needs to be slightly above 2 (for a temp of 34°C) and the stir dial should be ~ 5-6.
3. PBS:
  1. Prepare sterile PBS and fill 15 sterile 15ml tubes with 4-6 ml (be sure to maintain sterile technique).
  2. Place the tubes and the stock bottle on ice next to the hood.
4. Carbogen:
  1. Break a 5ml stripette in half and push it through a rubber stopper, so the tip of the stripette is sticking out the wide end of the stopper.
  2. Place the stopper in a 500ml flask with 400-500ml dH_2_O (broken end of stripette should be IN the dH_2_O).
  3. Connect a tube on the side opening of the flask.
  4. Cut the tip off a yellow pipette tip and connect this to the end of the tube (with the cut side facing out).Connect the carbogen tank to the tip of the 5ml stripette.
5. Prepare papain sol’n:
  * Use careful sterile technique.
  2. Mix in a sterile 50ml tube.
6. Filter papain sol’n into the dissociation vial:
  1. Use a Steriflip filtration unit and transfer to dissociation vial via 25ml stripette (OR place a new 0.2μm syringe filter on the tip of a 20ml plunger syringe, remove the plunger and use a stripette (25mL) to transfer all of the papain sol’n into the syringe.
  2. Filter into the dissociation vial).
  3. Cap the vial, insert a sterile syringe through the hole in the cap and attach a sterile 0.2μm syringe filter to the base of the syringe.
  4. Connect the carbogen to the filter and parafilm this in place. Place the vial in the Styrofoam disc/holder.*The micro-magnetic stir bar must be moving. *The gas MUST be making it into the vial. *The temperature MUST stabilize at 34°C.
7. Dissection prep:
  1. Bring the dissection microscope under the hood.
  2. Lay out all dissection instruments on aluminum foil, spray with 70% EtOH, pour off excess and leave under the hood to dry.
8. Prepare neuronal medium:
  1. Leave 30ml of fresh neuronal medium in the incubator.
9. Lay out 1 large square of aluminum foil and 12 smaller squares. Leave the decapitation scissors with the aluminum foil. Fill a small Styrofoam box with ice water and leave all outside of the hood.
10. Prep the anesthesia (0.075ml ketamine and 0.075ml xzylazine).
11. Get at least 12 pups (P0-P2) for 100 plates. For mice, make sure there is a genotype match for the whole litter. If genotype is unknown, plate cells from each pup on a separate dish, keep accurate records of mouse numbers and match them with cell culture dishes and make sure the genetic background of the glial substrate is uniform across cell cultures.

#### 2) Dissection

1. Anesthetize the first animal with an intraperitoneal injection. When animal shows sedation and does not respond to tail-flick test; put it in ice for 30 seconds (until hypothermic). Rinse one small aluminum foil square, decapitation scissors, and head with 70% EtOH.
2. Decapitate, allow head to fall onto aluminum foil square and move under the hood. Gently remove brain into a 15ml tube of ice-cold PBS. Place the tube back on ice while removing the next brain.
3. Repeat these first steps until there are 3 brains on ice. Pour all 3 brains and PBS onto the first dissection dish under the microscope. Remove appropriate section of the brain (VTA) and use a transfer pipet to put the segments into papain sol’n. Start a timer when the first segments go in.
4. Repeat previous 3 steps until all brains are being digested in papain. The average time for digestion should be 2 hours. The first segments will have been in for about 1 hour by the time the last ones go in. Try to split the difference.
*During digestion:
  1. Make sure the temp in the bath is 34°C.
  2. Segments may stick to the stir bar at first, but should spread out as time goes on. If they do not, tap the bottom of the vial.

#### 3) Trituration:

1. Using a transfer pipette, transfer the brain segments to a sterile 15ml tube (try to avoid excess papain sol’n) and WASH them 3 times with 2ml of warmed glial medium from the incubator. After putting the glial media in the tube, allow segments to settle and then carefully remove as much sol’n as possible without disturbing the segments. Change pipettes to avoid contamination! **DO NOT KEEP the sol’n that is removed.
2. Using a fresh transfer pipette begin triturations in 2ml of glial media. Triturate 25 times (avoid letting in air bubbles) and let the tube sit for 3 minutes until undissociated segments settle. Use a transfer pipette to remove and KEEP as much sol’n as possible without disturbing segments at the bottom. Keep the supernatant in a new (sterile) 15m tube.
3. Repeat the previous step using a 1000μl pipette and 200μl pipette until completely dissociated.
4. Triturate 10 times with a transfer pipette, or until the cells are completely resuspended in sol’n.

#### 4) Plating Cells

1. Using a sterile yellow pipette tip covering each tip of the forceps, carefully drop a slide ring as centered to the glass as well as possible inside each dish.
2. Put 10μl of the cell solution on the hemacytometer and count. (Exclude junk masses).
3. Multiply the number of cells by 10. This is the number of cells/μl. Divide 1,000,000-1,500,000 (or desired density) by this number. This is the number of μl to add per dish.
4. Use the pipette tip to slide rings over the center-well of each dish as you add appropriate μl/dish. Plate them gently and switch tips for EVERY dish.
5. Add 100μl (of diluted) GDNF sol’n inside the slide ring of each dish.
6. At this time, place the trays in the incubator and bring the neuronal medium in the cold room.
7. Allow cells to settle overnight.
8. Next day: remove rings (using new sterile pipette tips covering the forceps tips for EVERY dish)

#### 5) Mitotic Inhibition: THE NEXT DAY

1. Dilute FDU aliquots of 1000X stock 1:10 by adding 200μl FDU stock to 1.8 ml sterile MEM.
2. Add 20μl diluted FDU sol’n to the outside ring of each dish. Change pipette tips often. Cover and return to the incubator. *Disturb the dishes as little as possible for the next 7-10 days. Check for infected dishes and remove any infected dishes at the first signs of infection. Cultures are ready for testing within 3 weeks.

 

### MEDIA/SOLUTIONS

**Neuronal Medium**(for 200mL)

**Best if conditioned on glia from flasks overnight before use for washes/trituration

**Table:** 

**Ingredient**	**Amount**	**Notes**
BSA 5%	0.5 g	Fraction V
MEM liquid	94.0 ml	Sigma
DMEM liquid	80.0 ml	Sigma
F-12 liquid	20.0 ml	Sigma
Glucose 45% liquid	1.50 ml	Sigma solution
Glutamine 200mM	0.5 ml	Aliquotted Sigma solution
Diporzio Conc.	2.0 ml	Sigma solution
Liquid Catalase	0.1 ml	
Kynurenic acid 0.5M	200μl	In 1N NaOH
HCL 5N	50 μl

1. Combine ingredients (BSA goes last) in a 250ml plastic bottle.
2. Filter, label, and refrigerate.

**** 

**Kynurenic Acid**(FW = 189.2) 0.5M = 94.6mg/ml Make 8ml stock: 756.8mgKA/8ml 1N NaOH and pipette into STERILE aliquots of 200μl

**DiPorzio Media**

**A) DiPorzio Conc. Stocks:**

**Table:** 

**NEED**	**Combine**	**Alliquot**
**Additive**	** Solvent**	** Tube**	** Amount**	** ml**	** ml/tube**	** Conc.**	** Amount**	** # Aliq.**
Insulin	20mM HCL (1)	plastic	250mg bottle	10	1	25mg/ml	25mg	10
Transferrin	Hank’s		500mg bottle	5	1	100mg/ml	100mg	5
SOD	Hank’s		70mg bottle	14	1	5mg/ml	5mg	14
Putrescine	Hank’s		50mg	3	0.12	20mg/ml	2.4mg	21
Na_2_SeO_3_	Hank’s		0.104mg	10	0.5	10μg/ml	5.2μg	20
T3	10mM NaOH		2mg	10	0.1	0.20mg/ml	0mg	100
Progesterone	100% EtOH	Glass	12.5mg	10	0.05	1.25mg/ml	Use pipet	
Cortisol	100% EtOH	Glass	20mg	10	0.02	2.00mg/ ml	Use pipet

1. 20mM HCl = 41.5μl conc. HCl/25ml.
2. Make 1mg/ml stock and add 104μl to 10ml.

**B) DiPorzio Media Stock:**

**Table:** 

** Additive**	** Amount (ml)**	** Amount (ml) X2**	** Final Conc. μg/ml**	**Final Conc. Molarity**
Progesterone	0.05	0.1	0.06	200nM
Cortisol	0.02	0.04	0.04	125nM
Hank’s BSS	6.21	12.42		
Insulin	1	2	25	
Na_2_SeO_3_	0.5	1	0.01	30nM
T3	0.1	0.2	0.02	30nM
SOD	1	2	5	
Putrescine	0.12	0.24	2.4	15nM
Transferrin	1	2	100

1. Use a 15ml polyproylene sterile tube to add the progesterone and cortisol. Use an aspirator pipet and vacuum to speed evaporation of EtOH: (use a 5ml stripette broken in half and place half-way down into the tube being very careful not to aspirate EtOH liquid. Hold pipet securely in place with Kimwipes and then bring the tip down to the 500μl mark located on the side of the tube.
2. Add the subsequent aliquots in the order they appear on the above chart.
3. After the addition of insulin, which makes the sol’n cloudy, add 20μl of 1N NaOH to neutralize the pH. Sol’n should go from yellow to pink and immediately clear. ALSO, after the addition of transferrin, immediately add 20μl of 1N NaOH, to neutralize the pH and prevent the formation of precipitates.
4. Draw up 10ml into a serological pipet and divide into 5 aliquots (one batch).
5. Store @ -20°C.
6. May make 2 batches at once.

 

**Dissociation Media** **A. Papain (for 1 vial):**

**Prepare day of plating right before dissection.

**Table:** 

**Ingredient**	**Amount**	**(Notes) Final Conc.**
Cysteine Water	7.8mL	1mM cysteine
Papain	Varies (*190μl for the bottle of 44.0mg/ml)	20 units/ml (400 units total for 20ml worth of sol’n)
H&B conc.	2mL	
Carbogen	95% O2 + 5% CO2	
HCl 5N	10μl	
0.5% Phenol red	20μl	0.001%
Kynurenate 0.5M	10μl	(In 1N NaOH) 0.5mM

1. Add papain to the cysteine water FIRST.
2. Add the H&B conc., kynurenate, HCl, and phenol red.
3. Filter into dissociation vial (as described in the set-up directions) and connect to the carbogen.

**B. H&B Concentrate (100ml of 5X):**

**Table:** 

**Ingredients **	**M.W. **	** Powder/50ml H_2_O **	**Conc. (M) **	**Combine (ml) **	** Final Conc. (mM)**
NaCl	58.44	11.699 g	4	14.5	116
KCl	74.56	3.728 g	1	2.7	5.4
NaHCO_3_	84.01	4.2 g	1	13	26
NaH_2_PO_4_*H_2_O	137.99	6.90 g	1	1	2
MgSO_4_	120.38	6.019 g	1	0.5	1
EDTA (ED2- SS)	292	Sigma 5% (make 5g/100ml stock)	0.134	0.3722	0.5
Glucose	180	Sigma 45%	2.5	5	25
TC H_2_O				62.93

1. Combine amounts from stock solutions.
2. Divide into 4ml aliquots.
3. Add aliquot to cysteine water after adding papain.****

**C. Cysteine Water (157.5ml of 1X):**

****

**Table:** 

**Ingredients **	**M.W. **	** ComponentsPowder (mg)**	**H_2_O (ml)**	** Stock Conc.(mM) **	**Combine (ml)**	**Final Conc. (mM)**
CaCl_2_	147.2	736	10	500	0.6	1.9mM
Cysteine	121.7	(1.5)	24	20mM (0.02M)	10	1.27mM (0.88)
TC water					146.9

1. Make and keep a stock sol’n of 0.5M CaCl2 at 4°C
2. Make a one use sol’n of cysteine and combine with other ingredients
3. Divide into 10 aliquots of 15.75 ml and store at 4°C

**GDNF Preparation**

1. Aliquot prep:
  1. Dissolve sterile, lyophilized pellet of 5μg GDNF with 2.4mL of sterile deionized water. The concentration of this GDNF sol’n = 2.08μg/mL.
  2. Distribute the 2.08μg/mL GDNF sol’n into 76.9μl aliquots in sterile cryovials. This = 160ng per vial.
  3. Store at -20°C.
2. Addition to cultures:
  1. Thaw 1 aliquot for every 8 dishes.
  2. Dilute aliquot by adding 723.1μl neuronal media/aliquot. This will make a sol’n of 160ng/800μl GDNF.
  3. Add 100μl of this sol’n to the 2mL of medium in each dish for a final concentration of 10ng GDNF per mL.

**FDU Preparation** **FDU-sol’n 1000X Stock:**

**Table:** 

**Ingredient**	**Amount**	**(Notes) Final Conc.**
Uridine	247 mg	16.5 mg/ml
5-FDU (5-fluorodeoxyuridine)	100 mg (bottle)	6.7 mg/ml
TC Water	15 ml

1. Make a little over 15 ml of 16.5 mg/ml uridine.
2. Add 15 ml uridine sol’n to 100 mg bottle of FDU to make 6.7 mg/ml sol’n FDU.
3. Divide into 200μl aliquots and freeze in -20°C.

Dilute for Use:

1. Inhibit the growth of non-neuronal cells. Dilute 1000X stock 1:10 by adding 200μl stock to 1.8 ml MEM.
2. Add 20μl diluted FDU to the outside ring of each dish. Change pipet tips often. Cover and return to the incubator.

## Discussion

The methods described here allow for fine resolution of morphological and neurochemical features of central dopamine neurons that are otherwise not available in a systemic or *in vivo* approach.
